# Discovery of a FLT3 inhibitor LDD1937 as an anti-leukemic agent for acute myeloid leukemia

**DOI:** 10.18632/oncotarget.23221

**Published:** 2017-12-14

**Authors:** Hyo Jeong Lee, Jungeun Lee, Pyeonghwa Jeong, Jungil Choi, Juhwa Baek, Su Jin Ahn, Yeongyu Moon, Jeong Doo Heo, Young Hee Choi, Young-Won Chin, Yong-Chul Kim, Sun-Young Han

**Affiliations:** ^1^ College of Pharmacy and Research Institute of Pharmaceutical Sciences, Gyeongsang National University, Jinju, Republic of Korea; ^2^ School of Life Sciences, Gwangju Institute of Science and Technology, Gwangju, Republic of Korea; ^3^ Biomedical Science and Engineering, Gwangju Institute of Science and Technology, Gwangju, Republic of Korea; ^4^ Gyeongnam Department of Environment Toxicology and Chemistry, Korea Institutes of Toxicology, Jinju, Republic of Korea; ^5^ College of Pharmacy and BK21PLUS R-FIND Team, Dongguk University-Seoul, Goyang, Republic of Korea

**Keywords:** FLT3, indirubin, acute myeloid leukemia, anti-tumor agent

## Abstract

FMS-like receptor tyrosine kinase-3 (FLT3) belongs to the family of receptor tyrosine kinase (RTK), and the FLT3 mutation is observed in 1/3 of all acute myeloid leukemia (AML) patients. Potential FLT3 inhibitors have been investigated as potential therapeutic agents of AML. In this study, we identified a potent FLT3 inhibitor LDD1937 containing an indirubin skeleton. The potent inhibitory activity of LDD1937 against FLT3 was shown with an *in vitro* kinase assay (IC_50_ = 3 nM). The LDD1937 compound selectively inhibited the growth of MV-4-11 cells (GI_50_ = 1 nM) and induced apoptotic cell death. LDD1937 caused cell cycle arrest at the G_2_/M phase and increased the cell population at the sub-G_1_ phase. Phosphorylation of STAT5, which is the downstream signaling of FLT3, was significantly reduced by LDD1937 in a dose-dependent manner. The pharmacokinetic properties of LDD1937 were investigated in mice. Then, the *in vivo* anti-tumor effect was investigated using a MV-4-11 xenograft. With the intravenous administration of 5 and 10 mg/kg in nu/nu mice, the tumor volume and weight were significantly reduced compared to the control. LDD1937 is a promising therapeutic candidate to treat AML patients because of its ability to suppress tumor cell growth *in vitro* and *in vivo*.

## INTRODUCTION

FMS-like receptor tyrosine kinase-3 (FLT3), a receptor tyrosine kinase (RTK) of the type III RTK family, has an important role in the survival and proliferation of hematopoietic cells [1]. The activation of FLT3 is initiated by the binding of the FLT3 ligand, which is expressed by stromal cells, to the receptor. As a result, FLT3 receptor dimerization and auto-phosphorylation trigger the downstream signaling pathways, which are categorized into the PI3K/AKT, RAS/MAPK, and STAT5 signaling pathways [2]. The FLT3 receptor is expressed at high levels in 70–100% of AML cells. The significance of FLT3 in leukemia has been thoroughly investigated, and the population of the FLT3 mutations was reported to be approximately 1/3 of all AML patients [3]. Two major types of FLT3 mutations have been identified: internal tandem duplication (ITD) mutations in the juxtamembrane region and point mutations in the kinase domain [4]. These mutations confer an adverse prognostic influence with chemotherapy failure and relapse [5–8]. Moreover, recent studies have shown that FLT3-ITD mutations represent a driver mutation for the progression of AML consequently making them valid therapeutic targets in AML [9, 10]. Therefore, many researchers and pharmaceutical companies have tried to find FLT3 inhibitors as potential therapeutic agents of AML.

Several clinical candidates targeting FLT3 have been reported including lestaurtinib [11], tandutinib [12], sorafenib [13], KW-2449 [14], and quizartinib [15]. Among them, lestaurtinib is indolocarbazole derivative and well known multi-targeted tyrosine kinase inhibitors. A piperazinyl-quinazoline compound, tandutinib, inhibits FLT3 as well as c-Kit and PDGFR. Most of these inhibitors were redirected to AML by the inhibition of the FLT3-ITD mutation from their initial purpose of targeting other kinases. Although a FLT3 inhibitor developed by Novartis midostaurin (Rydapt^®^) was recently approved by the FDA [16], it seems that most of the current FLT3 inhibitors are unimpressive mainly because of their potency and target selectivity [17–20]. Therefore, the development of new potent and selective FLT3 kinase inhibitors is needed at the present time.

Our group previously reported that indirubin analogues potently inhibit FLT3 kinase [21]. After further development of the indirubin derivatives and their inhibitory activity, we identified a novel FLT3 inhibitor, LDD1937, through a kinase inhibitory assay of synthesized compounds, which significantly inhibited the growth of an AML cells. The ability of LDD1937 to suppress tumor cell growth *in vivo* and *in vitro* makes it a promising candidate to treat AML patients as well as to possibly treat other types of cancers also.

## RESULTS

### LDD1937 is an inhibitor of the FLT3 kinase activity

We previously reported that a series of 5-substituted indirubin derivatives are potent FLT3 inhibitors [21], which effectively inhibited the growth of acute myeloid leukemic cells. While the indirubins had a potent kinase inhibitory activity, their poor solubility in water caused some physiological problems. To address the solubility problems of these indirubin derivatives, in this study, we designed and synthesized new analogues with hydrophilic functional groups on the molecules.

**Figure 1:**
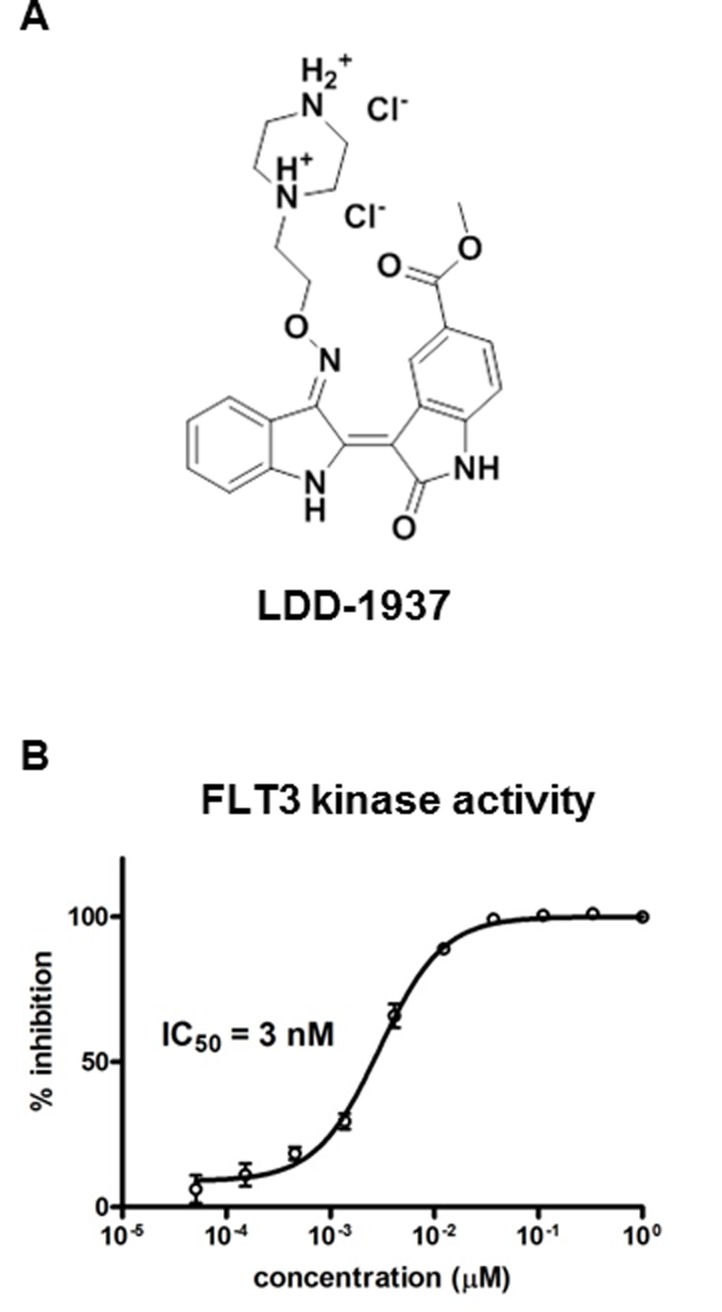
Structure of LDD1937 and its inhibitory effect on the FLT3 kinase activity (**A**) Chemical structure of LDD1937 (**B**) Effect of LDD1937 on the *in vitro* FLT3 kinase activity. Inhibition of kinase activity of recombinant FLT3 was measured with the HTRF assay. Kinase inhibition was calculated with 1% DMSO as a negative control. Data are the mean ± SEM of three independent experiments.

Several indirubin analogues were synthesized, and their structure activity relationship was investigated (Supplementary Table 1). Among 13 compounds, the LDD1937 compound (Figure 1A), methyl (2Z,3E)-2′-oxo-3-((2-(piperazin-1-yl)ethoxy)imino)-[2,3′-biindolinylidene]-5′-carboxylate dihydrochloride, was selected and further characterized. As shown in Figure 1B, the IC_50_ of LDD1937 against the FLT3 kinase activity was 3 nM. The IC_50_s against other kinase activities were also measured (Table 1). There was at least a 170-fold difference in the IC_50_ between FLT3 and the other kinases.

**Table 1:** *In vitro* activity of LDD1937 against select kinases

Kinase	IC_50_ (µM)
FLT3	0.00300 ± 0.000525
JAK2	0.523 ± 0.0727
JAK3	0.690 ± 0.0599
cMET	0.239 ± 0.0740
IRAK4	0.300 –

Data indicate mean ± S.D.

### LDD1937 selectively suppresses the cell growth of MV-4-11 cells and induces apoptotic cell death

MV-4-11 cells are leukemic cells with a FLT3 kinase mutation. The MV-4-11 cells harbor a FLT3 mutation with an internal tandem duplication (FLT3-ITD) in the juxtamembrane domain, which causes the constitutive activation of the FLT3 activity [3]. MV-4-11 cell growth and survival are known to be dependent on the FLT3 activity [22]. The cytotoxicity by LDD1937 was measured and shown in Table 2. Immortalized T lymphocytes Jurkat cells, prostate cancer PC-3 cells, breast cancer MCF-7 cells, and erythroleukemia K562 cells were also subjected to the cytotoxicity assay. The MV-4-11 cells exhibited a high sensitivity to the LDD1937 treatment (GI_50_ = 1 nM) compared to the other cell lines. In terms of the GI_50_ value, LDD1937 was 1,000 ∼ 2,000 times more potent in the MV-4-11 cells than in the other cell lines.

**Table 2:** Anti-proliferative activities of LDD1937 against various cancer cell lines

Cell line	GI_50_ (µM)
MV-4-11	0.0012 ± 0.0002
Jurkat	1.44 ± 0.29
PC-3	1.1 ± 0.14
MCF-7	2.14 ± 0.16
K562	1.21 ± 0.33

Data indicate mean ± S.D.

**Figure 2:**
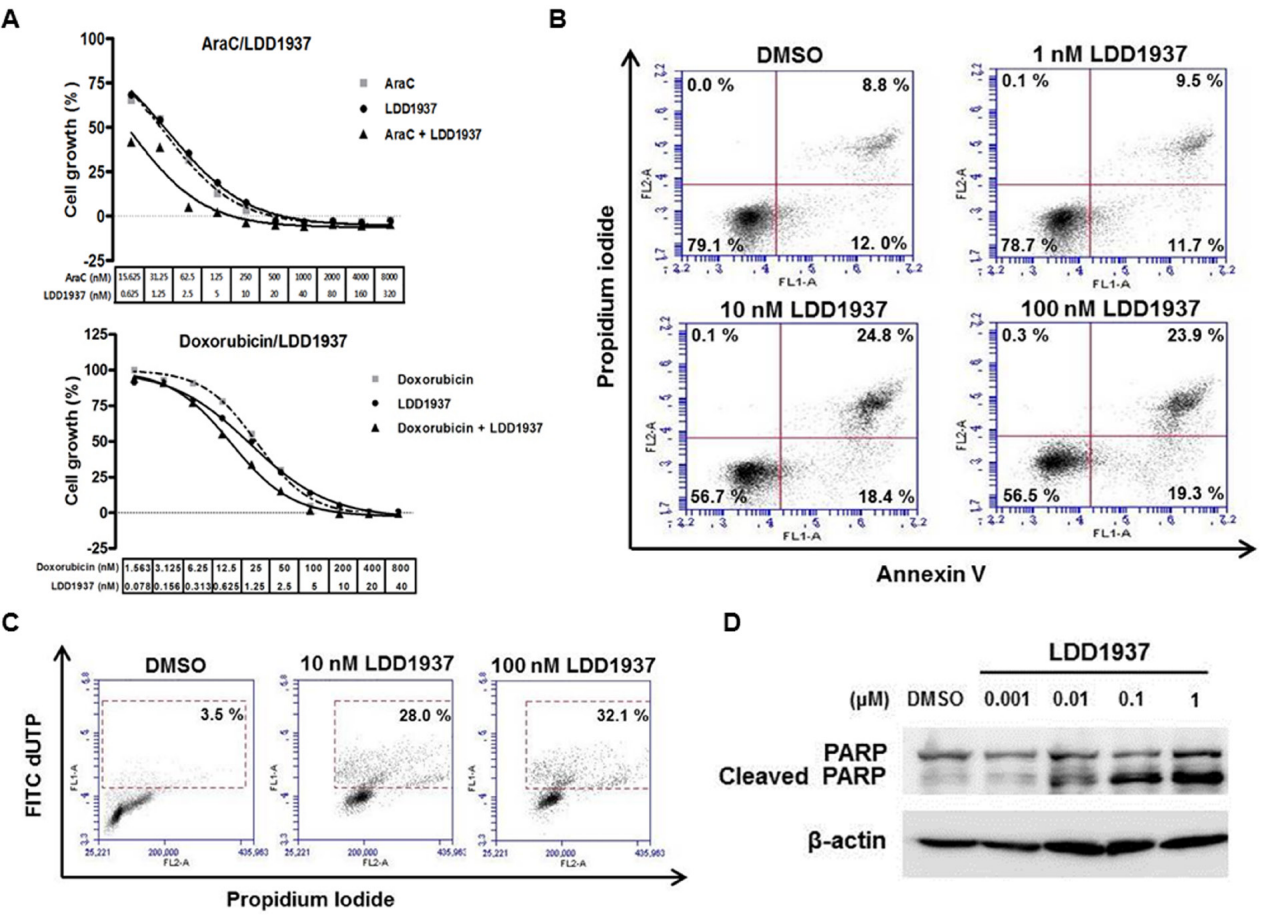
Effect of LDD1937 on the MV-4-11 cell growth and cell death (**A**) MV-4-11 cells were treated with AraC, doxorubicin, or LDD1937 either alone or in combination at a fixed dose ratio of AraC vs. LDD1937 (ratio 25:1) and doxorubicin vs. LDD1937 (ratio 20:1) for 72 h at the indicated concentration, and the cell growth was measured. The CI value was calculated with the CompuSyn software. (CI < 1: synergistic; CI = 1: additive; CI > 1: antagonistic) (**B**) MV-4-11 cells were treated with LDD1937 at the indicated concentration for 24 h. The cells were stained with propidium iodide/Annexin V, and flow cytometry was performed. Bottom right quadrant (Annexin V+ and PI−): early apoptotic cells: top right quadrant (Annexin V+ and PI+): late apoptotic and already dead cells. (**C**) MV-4-11 cells were treated with LDD1937 for 24 h, and the TUNEL assay was performed as described in the Material and methods. The TUNEL positive population is indicated in the box with a dotted line. (**D**) MV-4-11 cells were treated with LDD1937 for 24 h, and PARP cleavage was measured by western blot analysis using an antibody that detects both the cleaved and uncleaved PARP.

AraC (cytarabine or cytosine arabinoside) and doxorubicin (Adriamycin^®^) are the anticancer drugs used for standard chemotherapy for AML patients [23]. To investigate the possibility of combination therapy using LDD1937 and AraC/doxorubicin, the combination indexes (CI) of LDD1937/AraC and LDD1937/doxorubicin were measured. As shown in Figure 2A (upper panel), AraC and LDD1937 were treated at a ratio of 25:1, and then, the MV-4-11 cell growth was measured. The calculated CI value at GI_50_ was 0.160 (less than 1) indicating a synergism between LDD1937 and AraC. Doxorubicin and LDD1937 were treated at a ratio of 20:1, and the CI value was calculated as 1.06 indicating an additive effect between LDD1937 and doxorubicin (Figure 2A, lower panel).

Apoptotic cell death by LDD1937 was measured with annexin V staining. The apoptotic cell population increased in a dose-dependent manner shown in Figure 2B. Late apoptotic cells (right upper quadrant of the annexin V/PI staining graph) changed from 8.8% (DMSO control) to 24.8% (10 nM). The TUNEL assay also confirmed the apoptosis by LDD1937 (Figure 2C). The TUNEL positive cell population increased from 3.5% (DMSO control) to 32.1% (100 nM). Western blot analyses of the cleaved PARP also showed that the LDD1937 treatment induced PARP cleavage in a dose-dependent manner. With a 1 nM LDD1937 treatment, an increase in the cleaved PARP was detected using an antibody that binds both uncleaved and cleaved PARP.

### LDD1937 induces cell cycle arrest

**Figure 3:**
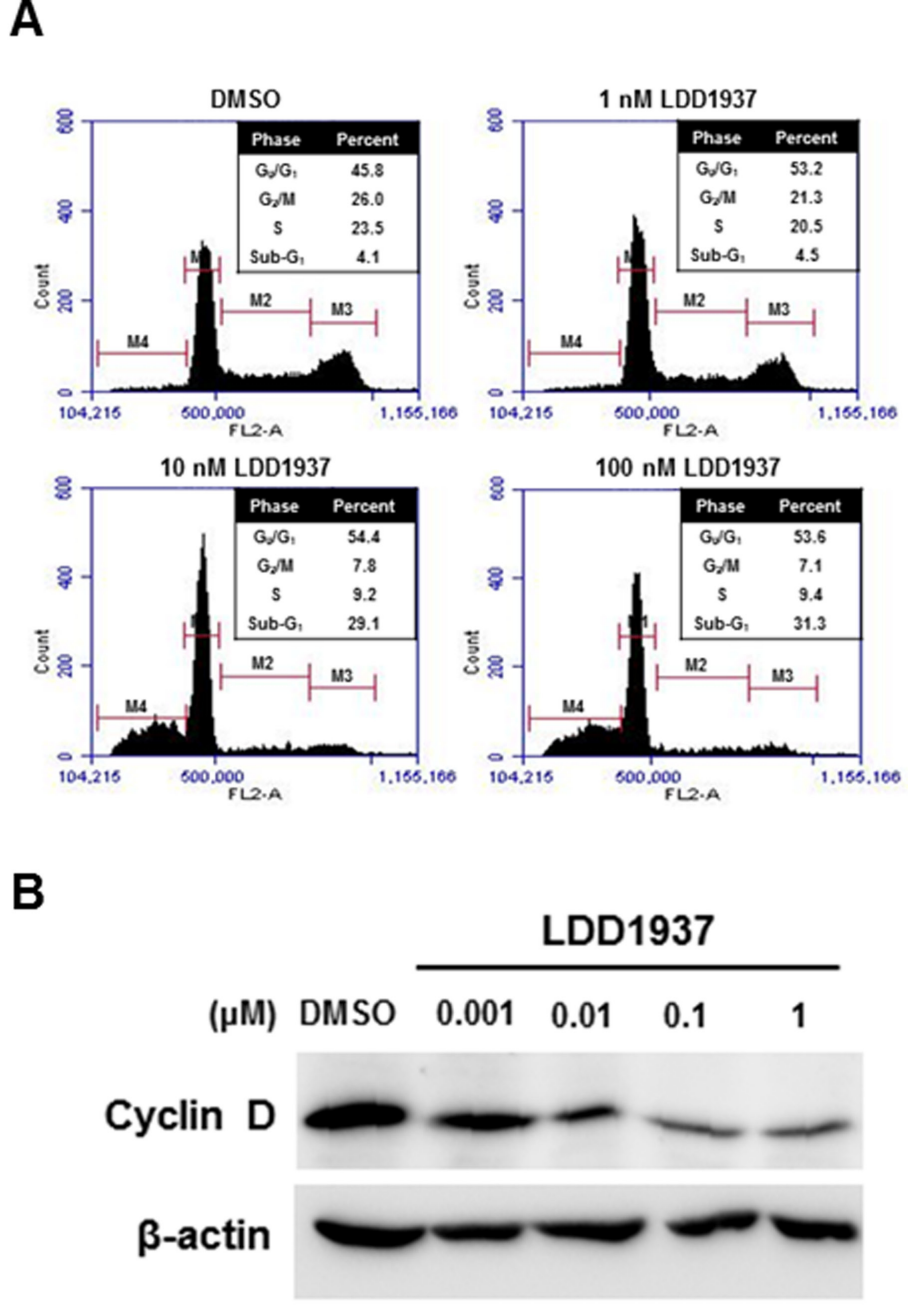
Effect of LDD1937 on the MV-4-11 cell cycle (**A**) MV-4-11 cells were treated with LDD1937 for 24 h and stained with PI. Cells were then subjected to cell cycle analyses using a flow cytometer. M1: G_1_ phase, M2: S phase, M3: G_2_/M phase, M4: sub-G_1_ phase. (**B**) MV-4-11 cells were treated with LDD1937 at the indicated concentration for 16 h. Cyclin D1 levels were detected by western blot. As a loading control, the western blot of β-actin was done.

Cell cycle distribution was assessed by flow cytometry after treating the MV-4-11 cells with LDD1937 (Figure 3A). The LDD1937 treatment induced a significant change in the cell cycle populations. The proportion of cells in the G_0_/G_1_ phase slightly increased from 45.8% (DMSO control) to 54.4% (10 nM). Consistent with the cell cycle arrest at the G_0_/G_1_ phase, the expression of cyclin D1 decreased with the LDD1937 treatment. A dose-dependent reduction of the cyclin D level was observed from as low as 10 nM of LDD1937 (Figure 3B). The sub-G1 population indicating the dead cell population increased by the LDD1937 treatment from 4.1% (DMSO control) to 31.3% (100 nM). The results in Figure 3 are consistent with the cell growth suppression (Table 2) and apoptosis of the MV-4-11 cells (Figure 2).

### LDD1937 inhibits the FLT3 signal transduction pathway

**Figure 4:**
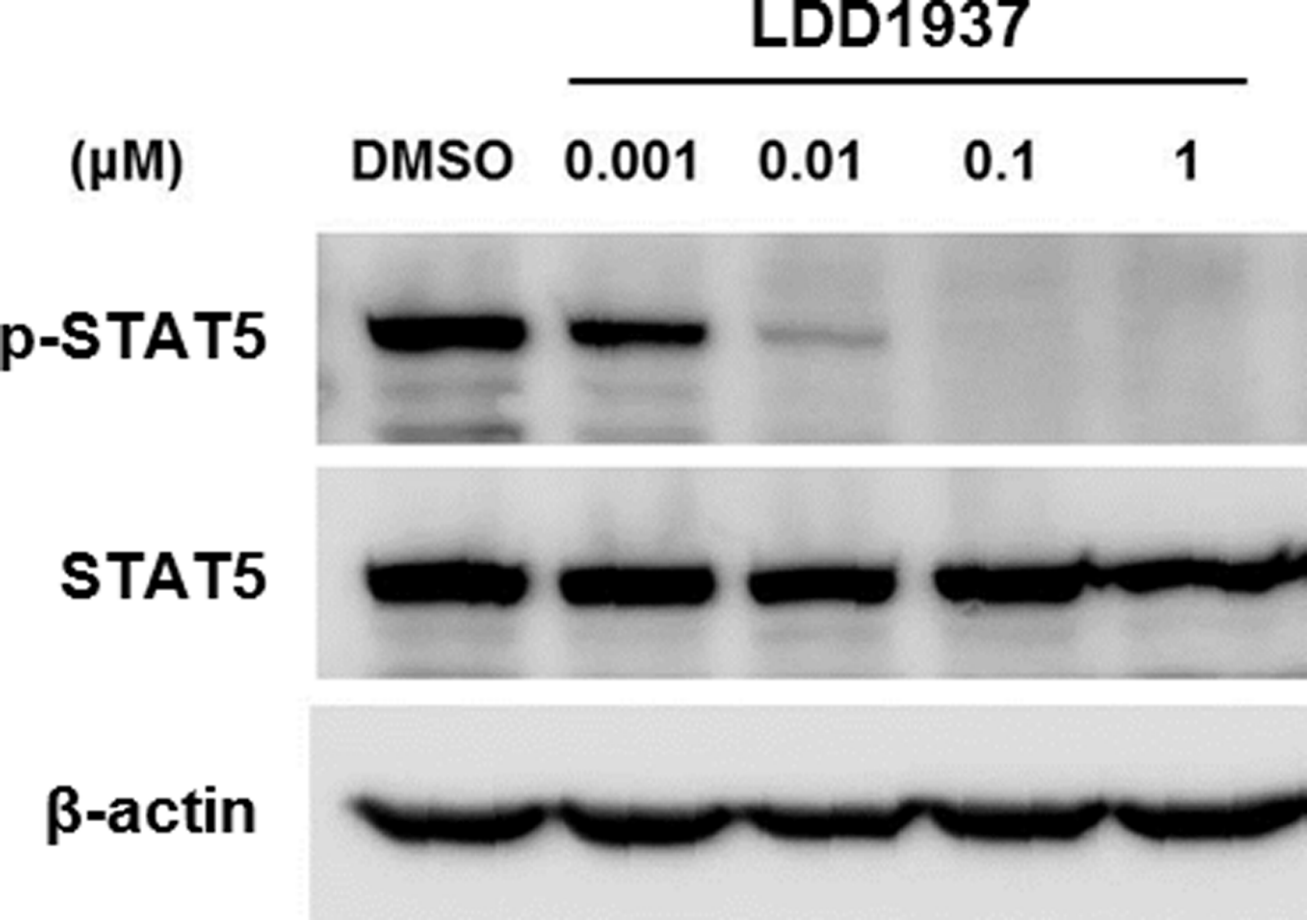
Effects of LDD1937 on the STAT5 phosphorylation MV-4-11 cells were treated with LDD1937 for 4 h at the indicated concentration. Cell extracts were subjected to western blot using an antibody against phosphorylated STAT5 (p-STAT5) and STAT5. As a loading control, western blot of β-actin was done.

The ability of the LDD1937 compound to inhibit the downstream signal transduction pathway was assessed by western blot analysis. The phosphorylation of STAT5 is an important part of the FLT3 signaling pathway [24], and treatment with the LDD1937 compound significantly reduced the phosphorylation of STAT5 at various concentrations. With 0.1 µM of LDD1937, the phosphor-STAT5 band completely disappeared (Figure 4). Moreover, a reduction in the phosphorylated STAT5 was observed with as low as 1 nM of LDD1937 treatment.

### Investigation of the LDD1937 pharmacokinetics

**Figure 5:**
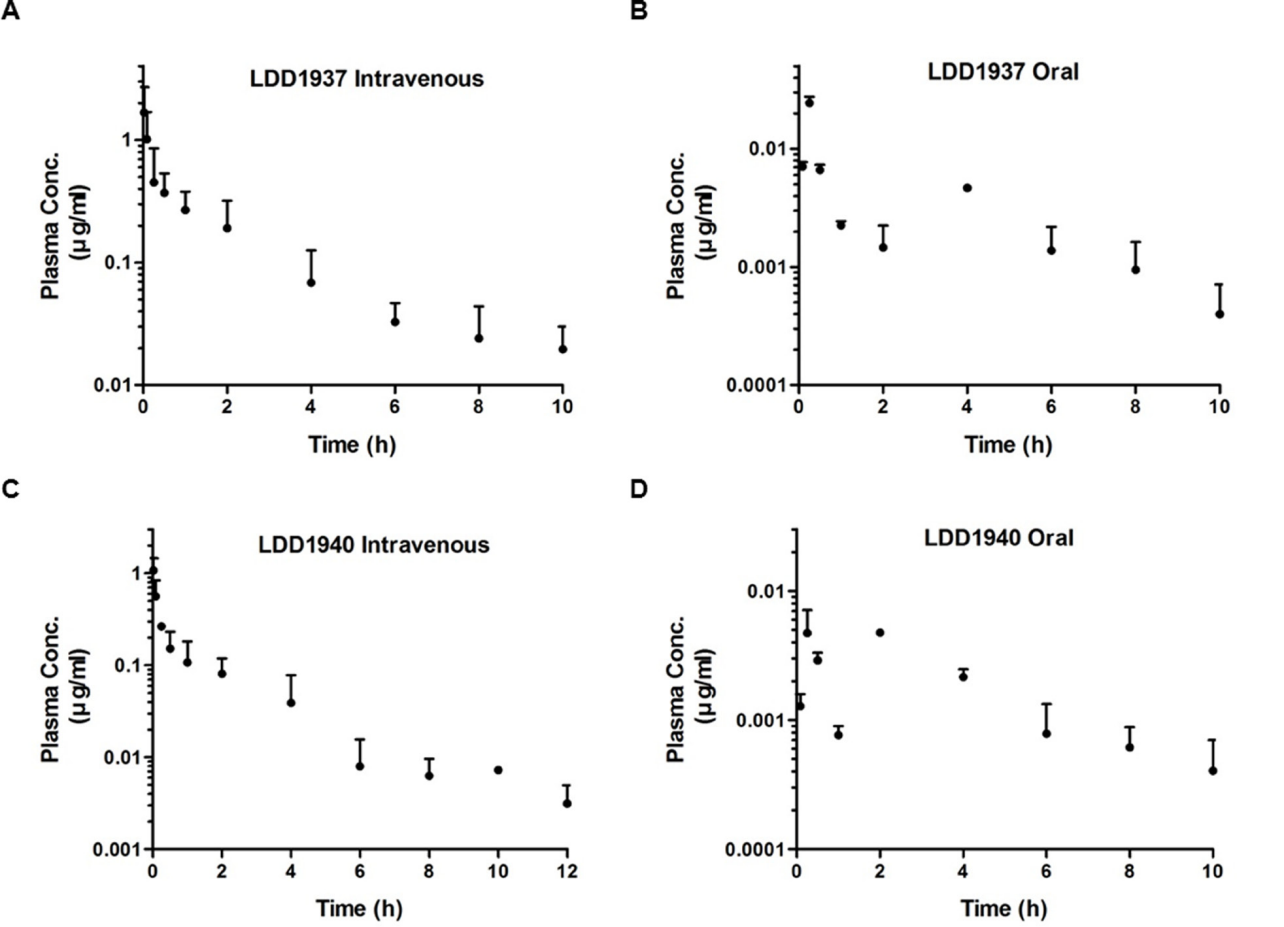
Pharmacokinetic study of LDD1937 10 mg/kg of the LDD1937 compound was intravenously injected into the mice through the tail vein (**A**, **C**) or administered orally (**B**, **D**). Blood samples were collected at the indicated time points after the injection. LDD1937 and LDD1940 in the blood samples were determined by LD-MS/MS analysis as described in the Material and methods. The data represent the mean ± SD.

The pharmacokinetic property of LDD1937 was investigated. The mean plasma concentration–time profiles of LDD1937 after intravenous and oral administration of LDD1937 in mice are shown in Figure 5A and 5B. The relevant pharmacokinetic parameters are listed in Table 3. The absorption of LDD1937 seemed to be almost complete because a small percentage of oral LDD1937 in the gastrointestinal tract (GI_24h_), 1.67%, was a sum of the percentage of unabsorbed and excreted LDD1937. This result is consistent with the result of the parallel artificial membrane permeability (PAMPA) assay. The PAMPA assay result for LDD1937 was –4.49 ± 0.140 indicating a medium level of permeability. Additionally, the non-renal clearance of LDD1937 was the main route of elimination due to the negligible contribution of the CL_R_ to CL, and the urinary excretion was 0.283% of LDD1937 in the intravenous study. Nevertheless, the *F* of LDD1937 was low, at 1.43% of the oral dose, indicating that extensive metabolism of LDD1937 might occur. Due to the low bioavailability, the intravenous route of administration was used for the *in vivo* xenograft study.

**Table 3:** Pharmacokinetic parameters of LDD1937 and LDD1940

Parameters	Intravenous		Oral
BW (g)	38.3 ± 1.35	BW (g)	31.9 ± 2.22
**LDD1937**		**LDD1937**	
AUC (µg min/ml)	120 ± 14.1	AUC (µg min/ml)	1.79 ± 0.689
Terminal half-life (min)	174 ± 28.2	Terminal half-life (min)	103 ± 32.5
CL (ml/min/kg)	83.6 ± 9.81	*C*_max_ (µg/ml)	0.0233 ± 0.0262
CL_NR_ (ml/min/kg)	83.6 ± 9.81	*T*_max_ (min)	30 (15–120)
MRT (min)	166 ± 32.0	*A*e_0–24 h_ (% of dose)	0.213 ± 0.0163
*V*ss (ml/kg)	14075 ± 4310	GI_24 h_ (% of dose)	1.67 ± 1.92
*A*e_0–24 h_ (% of dose)	0.283 ± 0.0252	*F* (%)	1.43
GI_24 h_ (% of dose)	0.336 ± 0.0235		
**LDD1940**		**LDD1940**	
AUC (mg min/ml)	54.6 ± 0.592	AUC (mg min/ml)	1.84 ± 0.0741
Terminal half-life (min)	142 ± 121	Terminal half-life (min)	742 ± 129
C_max_ (mg/ml)	1.45 ± 0.694	C_max_ (mg/ml)	0.00817 ± 0.00244
T_max_ (min)	1 (1–1)	T_max_ (min)	15 (15–120)
Ae_0–24 h_ (% of dose)	0.868 ± 0.0333	Ae_0–24 h_ (% of dose)	0.0787 ± 0.0791
GI_24 h_ (% of dose)	0.719 ± 0.0556	GI_24 h_ (% of dose)	0.792 ± 1.09
AUC_1940_/AUC_1937_ (%)	42.7 ± 9.44	AUC_1940_/AUC_1937_ (%)	65.3 ± 16.8

^a^10 mg/kg LDD1937 was administered to mice intravenously and orally. Data represent mean ± SD.

^b^Abbreviations: AUC, area under the curve; *A*e_0–24 h_, total amount excreted in 24-h urine; BW, body weight; CL time-averaged total body clearance; CL_NR,_ time-averaged nonrenal clearance; GI_24 h_, total amount recovered from the entire gastrointestinal tract (including its contents and feces) at 24 h; MRT, mean residence time; *V*ss, apparent volume of distribution at steady state.

Based on the chemical structure of LDD1937 as the ester form, hydrolysis of LDD1937 to carboxylic acid form (LDD1940) was expected. Therefore, the pharmacokinetic profile of LDD1940 was also explored (Table 3), and the mean plasma concentration–time profiles of LDD1940 after intravenous and oral administration of LDD1937 were calculated (Figure 5C and 5D). As expected, LDD1940 was formed fast with a Tmax of 1–50 min, after intravenous and oral administration of LDD1937. Additionally, the AUC1940/AUC1937 is 42.7−65.3% suggesting that LDD1940 is one of the main metabolites of LDD1937. Moreover, the urinary and biliary excretion of LDD1940 were low similar to LDD1937.

From the metabolite information acquired from the pharmacokinetic experiments, the major metabolite LDD1940 is expected to contribute to the anti-tumor effects of LDD1937. The FLT3-inhibitory activity (IC_50_ 2.45 nM) of LDD1940 is comparable to LDD1937 (Supplementary Table 1). A relatively strong growth inhibitory activity (MV-4-11 GI_50_ 40 nM) is shown by LDD1940 (Supplementary Table 1).

### LDD1937 suppresses tumor growth *in vivo*

**Figure 6:**
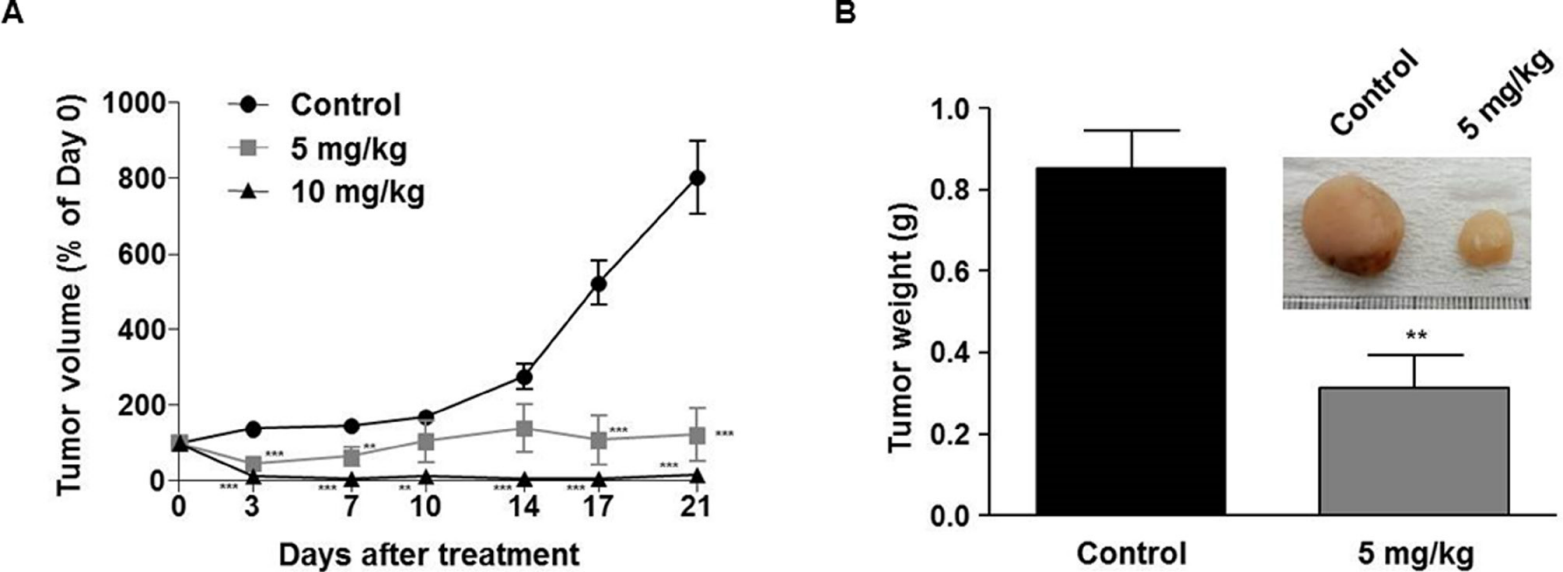
*In vivo* antitumor efficacy of LDD1937 MV-4-11 cells were inoculated subcutaneously into BALB/c *nu/nu* mice. When the tumor reached a mean volume of 100 mm^3^, mice were injected with 5 mg/kg or 10 mg/kg of LDD1937 or PBS (control) in the tail vein daily for 21 days. The tumor size was measured, and the tumor volumes were calculated (**A**). At day 21 after drug administration, mice were sacrificed, and the tumor weights measured (**B**). Representative image of a tumor mass dissected from the control and 5 mg/kg group was photographed (Inset). The data represent the mean ± SEM. ^**^P < 0.01, ^***^P < 0.001 compared to the control, respectively.

To examine the efficacy of LDD1937 *in vivo*, a MV-4-11 xenograft study was performed. MV-4-11 cells were injected into BALB/c *nu/nu* mice subcutaneously, and tumors were grown to a size of approximately 100 mm^3^. Then, LDD1937 or the PBS control was administered intravenously for three weeks. As shown in Figure 6A, the tumor sizes in the LDD1937 group were dramatically smaller than those of the control group. Particularly in the 10 mg/kg group, the tumor disappeared from day 3 which was based on the measured tumor volume (Figure 6A). Dissection of the tumor injection site confirmed the complete disappearance of the tumor mass in the 10 mg/kg group. Therefore, the tumor weight could only be measured in the control group and 5 mg/kg group, which showed a significant reduction in the 5 mg/kg group (Figure 6B). There was no significant difference in body weight between the groups during the administration period (Supplementary Figure 1).

## DISCUSSION

In this study, the indirubin derivative LDD1937 compound was characterized as a FLT3 inhibitor with anti-leukemic activity. LDD1937 exhibited a potent *in vitro* activity against FLT3 kinase (Figure 1B) and suppressed the proliferation of MV-4-11 cells harboring the FLT3 mutation (Table 2). The suppressive effect of LDD1937 on the FLT3 activity and on MV-4-11 cell growth was selective. Apoptotic cell death was induced by LDD1937 (Figure 2), and cell cycle arrest was observed (Figure 3). Signal transduction to the STAT5 pathway was suppressed by LDD1937 consistent with the inhibition activity of FLT3 (Figure 4). Based on the pharmacokinetic profile of LDD1937 (Figure 5), an *in vivo* xenograft study was performed. The tumor volume and weight were dramatically suppressed by LDD1937 (Figure 6) indicating the potential of LDD1937 as an antileukemic agent.

Combinatorial treatment of LDD1937 and AraC/doxorubicin, currently used a cytotoxic drug for AML, was explored (Figure 2A). One of the common regimens called “7 + 3” is comprised of AraC infusion for days 1 to 7 and doxorubicin treatment for days 1 to 3 [23], and LDD1937 may require combination therapy with AraC/doxorubicin. The combination effect of a cytotoxic drug and targeted agent should be evaluated because antagonism may exist between the two drugs. The combination index, CI, was measured using the principle based on Chou *et al.* [25]. Treatment of LDD1937 and AraC together showed a synergism in the cytotoxic effect on the MV-4-11 cells, and an additive effect was observed between doxorubicin and LDD1937. There results provide the possibility of a combination regimen using LDD1937 and AraC/doxorubicin.

As mechanisms of the anti-leukemic effects, apoptotic cell death (Figure 2) and cell cycle arrest (Figure 3) by the LDD1937 treatment was investigated in this study. Other potential mechanisms such as differentiation and cellular senescence were also explored. Differentiation of leukemic cells was evaluated with Wright-Giemsa staining. However, differentiated cells were not observed in LDD1937-treated cells (data not shown). Senescence-associated β-galactosidase assay (SA-β-gal) was carried out to measure cellular senescence. However, SA-β-gal did not change with LDD1937 treatment (data not shown), indicating senescence may not be involved as a mechanism of the anti-tumor effects of LDD1937.

The pharmacokinetic properties of LDD1937 were investigated (Table 3). Based on the low *F* value (1.43%) in the oral administration, the metabolism of LDD1937 was expected. The chemical structure of the presumed LDD1937 metabolite corresponds to LDD1940 (Supplementary Table 1), which is the hydrolyzed form of LDD1937. The pharmacokinetic profile of the metabolite LDD1940 was measured and is shown in Table 3. The Tmax of LDD1940 was 1∼15 min, after intravenous and oral administration of LDD1937 indicating that the formation of the metabolite occurs rapidly. The sum of the AUC for LDD1937 and LDD1940 was 42.7%∼65.3% suggesting that LDD1940 is one of the main metabolites of LDD1937. The plasma concentration–time profiles of LDD1940 after intravenous and oral administration of LDD1937 in mice are shown in Figure 5. The inhibitory effect of LDD1940 on the FLT3 kinase activity was measured, and the potency of the metabolite is similar to the parent compound LDD1937. The IC_50_ of LDD1937 was 3 nM (Figure 1B) and that of the LDD1940 was 2.5 nM (Supplementary Table 1). LDD1940 also had a selective anti-proliferative effect on the MV-4-11 cells (Supplementary Table 2). Therefore, these results suggest that LDD1940 may contribute to the anti-tumor effect of LDD1937 *in vivo*.

After a single dose of a 10 mg/kg intravenous administration of LDD1937 in mice, the plasma concentration of LDD1937 reached up to 2.7 µg/ml (Figure 5A), which is 2,000 folds above the IC_50_ for the FLT3 inhibition in the biochemical assay (Figure 1B). At 10 h after the dosing, the plasma levels were 30 ng/ml, which is high enough for almost complete inhibition of the FLT3 activity. These favorable pharmacokinetic properties may contribute to the effective anti-tumor activity *in vivo*. A once daily administration of 10 mg/kg LDD1937 made the tumor xenograft completely disappear from day 3 (Figure 6A).

Midostaurin, also known as PKC412, is currently the only FDA-approved FLT3 inhibitor drug. Its indication is newly diagnosed AML that is FLT3 positive, in combination with standard cytarabine and daunorubicin induction and cytarabine consolidation. Monotherapy of midostaurin for induction therapy is not an approved indication. The oral route of administration of 50 mg twice daily with food is recommended. In terms of clinical application, pulmonary toxicity and interaction with CYP3A4 inhibitor and inducer are major disadvantages of this drug. Midostaurin is a derivative of staurosporine, a pan-kinase inhibitor. Although midostaurin is the first known PKC inhibitor as its name PKC412 implies, it is called a multi-kinase inhibitor with its broad activity against kinases including FLT3, VEGFR1/2, PDGFRβ, and PKCα/β/γ with IC_50_s ranging from 80-500 nM [26]. Improvement of the kinase selectivity, overcoming adverse effects especially pulmonary toxicity, and the removal of the drug interaction mediated by CYP3A4 will result in a better drug than that of midostaurin.

A great deal of unmet medical needs exist for AML therapy because the complete response rate for the current therapy is 65% to 85% [23]. In patients older than 60 years of age, only 39% to 64% of the patients achieve a complete response [23]. Many approaches for developing novel therapies for AML are ongoing, such as antibodies against CD33, epigenetic targets, and T cell immunotherapy [27]. FLT3 targeting is still a promising approach to overcome the treatment failure of AML despite the insufficient clinical results from recent trials. Experiences from FLT3 inhibitor clinical trials have accumulated, and the follow-up analysis of the clinical data suggests that more effective FLT inhibitors are still required [28]. Here, we presented the LDD1937 compound which has great potency *in vitro* and *in vivo* for antileukemic activity. Further studies with the LDD1937 compound may lead to the incorporation of FLT3 targeted agents in new therapies for AML.

## MATERIALS AND METHODS

### Cell culture

MV-4-11 human acute myeloid leukemia cells were purchased from the American Type Culture Collection (ATCC, Rockville, MD, USA, CRL-9591), and the cells were cultured in IMDM medium (Sigma Co., St. Louis, MO, USA) supplemented with 10% fetal bovine serum, 1% penicillin/streptomycin and 4 mM L-glutamine (Life Technology, Grand Island, NY). Jurkat (human acute T lymphocytic leukemia, ATCC TIB-152) and K562 (human chronic myelogenous leukemia, ATCC CCL-243) cells were cultured in RPMI-1640 (Sigma Co.), and MCF7 (human breast adenocarcinoma, ATCC HTB-22) and PC-3 (human prostate adenocarcinoma, ATCC CRL-1435) cells were cultured in DMEM (Sigma Co.) medium supplemented with 10% fetal bovine serum and 1% penicillin/streptomycin. The cultured cells were incubated at 37°C with 5% CO_2_.

Cell viability was assessed with a tetrazolium-based assay using the EZ-Cytox Cell Viability Assay kit (DaeilLab, Korea). Briefly, 2,000 ∼ 15,000 cells were plated in 96-well flat bottom plates in 100 µl of medium. The next day, the cells were treated with compounds along with dimethyl sulfoxide (DMSO, Fisher, Waltham, MA, USA) as a negative control. Three days (72 h) after the addition of the drug, 15 µl of the EZ-Cytox kit reagent were added to each well of the 96-well plate and then incubated at 37°C in a humidified CO_2_ incubator for 4 h. After incubation, the optical density (OD) was measured at a wavelength of 450 nm with a Victor multilabel reader (Perkin Elmer, Waltham, MA, USA). The IC_50_ was calculated with nonlinear regression using Prism version 5.01 (GraphPad, La Jolla, CA, USA).

The drug combination effect was measured based on the principle in Chou’s article [25]. Cells were treated with each compound alone and a combination of two compounds. Cell viability was assessed as described above, and the combination index (CI) was calculated with the CompuSyn software version 1.0 (ComboSyn, Paramus, NJ, USA).

### Compounds

LDD1937, Methyl-(2Z,3E)-2’-oxo-3-((2-(piperazin-1-yl)ethoxy)imino)-[2,3’-biindolinylidene]-5′-carboxylate dihydrochloride, was designed and synthesized. The compound was dissolved in DMSO at a concentration of 10 mmol/L and stored at –20°C. The synthetic procedures for all the compounds are available in the supporting information (Supplementary Figures 2–5).

### *In vitro* kinase assay

The inhibition of the FLT3 kinase activity was measured with homogeneous, time-resolved fluorescence (HTRF) assays. Recombinant proteins containing the FLT3 kinase domain were purchased from Carna biosciences (Japan). Optimal enzyme, ATP, and substrate concentrations were established with the HTRF KinEASE kit (Cisbio, France) according to the manufacturer’s instructions. The FLT3 enzymes were mixed with serially diluted compounds and peptide substrates in a kinase reaction buffer (50 mM HEPES (pH 7.0), 500 μM ATP, 0.1 mM sodium orthovanadate, 5 mM MgCl_2_, 1 mM DTT, 0.01% bovine serum albumin (BSA), and 0.02% NaN_3_). After the addition of the reagents for detection, the TR-FRET signal was measured with a Victor multilabel reader (Perkin Elmer, Waltham, MA, USA). The IC_50_ was calculated with nonlinear regression using Prism version 5.01 (GraphPad). JAK2, JAK3, cMET, and RET *in vitro* kinase assays were also carried out using the HTRF assay kit.

The *in vitro* kinase assay for IRAK4 was done with the LANCE Ultra Kinase Activity Assay (Perkin Elmer) containing the ULight-p70S6K (Thr389) peptide (phosphorylation motif FLGF**T**YVAP). The assays consisted of the enzyme mixed with serially diluted compounds, 50 nM ULight-p70S6K (Thr389) peptide, and 500 μM ATP prediluted in kinase buffer (50 mM HEPES (pH 7.5), 10 mM MgCl_2_, 1 mM EGTA, 2 mM DTT, and 0.01% Tween20). Ten microliters of the total volume of the kinase reaction were added to the wells of a 96-well assay plate. The kinase reactions were incubated for 90 min. at 25°C and stopped by the addition of 10 mM EDTA. For the detection of the phospho-substrate, the Eu-anti-phospho-p70S6K (Thr389) antibody diluted in detection buffer was added to a final concentration of 2 nM, and the reactions were then incubated for 1 h at 25°C. The signal was measured on an EnVision multi-label reader.

### Flow cytometry

For Annexin V staining, the Alexa Flour^®^ 488 Annexin V/ Dead Cell Apoptosis Kit (ThermoFisher, USA) was used. Cells were seeded in 6-well plates (1 × 10^6^ cells per well) and treated with compounds for 24 h. The cells were stained with Annexin V and propidium iodide (PI) according to the manufacturer’s instruction. Then, the cells were subjected to flow cytometry using an Accuri C6 flow cytometer (BD Bio-sciences, San Jose, CA, USA). The data were analyzed with the BD Accuri C6 software.

For the terminal deoxynucleotidyl transferase dUTP nick end labeling (TUNEL) assay, the APO-DIRECT Kit (BD Biosciences, San Diego, CA, USA) was used. Briefly, 2 × 10^6^ cells were harvested and fixed in 2% formaldehyde/PBS solution for 30 min. on ice. The fixed cells were washed twice with PBS and stored in 1 ml of 70 % ethanol at –20°C until use. After washing with wash buffer, the cells were incubated in DNA Labeling Solution (reaction buffer, TdT Enzyme, and FITC dUTP) for 60 min. at 37°C. After labelling, the cells were washed twice with rinse buffer. The cells were incubated with 200 µg/ml RNase A and 5 µg/ml PI for an additional 30 min at room temperature. Annexin V/PI staining was measured with an Accuri C6 flow cytometer.

For cell cycle analysis, cells were seeded in a 10 cm plate (2 × 10^6^ cells per well) and treated with the LDD1937 compound. Then, the cells were fixed with ethanol and treated with RNase A (4 mg/mL) and stained with propidium iodide (PI) (Sigma Co). Cell cycle distribution was analyzed by flow cytometry with an Accuri C6 flow cytometer.

### Western blot analysis

Cells were lysed in SDS Lysis buffer (12 mM Tris-Cl, pH 6.8, 5% glycerol, and 0.4% SDS), and the protein concentrations were measured with the SMART BCA Protein Assay kit (iNtRON Biotechnology, Korea). The proteins were resolved with SDS-polyacrylamide gel electrophoresis followed by transfer to PVDF membranes (Millipore, Billerica, MA, USA) and incubated overnight with the appropriate antibodies. The antibodies used were as follows: phospho-STAT5 (p-STAT5; rabbit polyclonal IgG; #9351, 1:1,000), cyclin D1 (rabbit polyclonal IgG; #2922, 1:1,000), and PARP (rabbit polyclonal IgG; #9542, 1:1,000) from Cell Signaling Technology (Danver, MA, USA), STAT5 (rabbit polyclonal IgG; #sc-835, 1:1,000) from Santa Cruz (Santa Cruz, CA, USA), and β-actin (mouse monoclonal IgG; # A5441, 1:5000) from Sigma-Aldrich. Goat anti-rabbit IgG (#111-035-003; 1;5,000) and anti-mouse IgG (#115-035-033; 1:5,000) secondary antibodies were obtained from Jackson ImmunoResearch Laboratories, Inc. (West Grove, PA, USA).

### Pharmacokinetics

The protocols for the animal studies were approved by the Institute of Laboratory Animal Resources of Dongguk University, Seoul, South Korea (IACUC-2015-055-1). Five-week-old male Imprinting Control Region (ICR) mice were purchased from the Charles River Company Korea (Orient, Seoul, South Korea). Upon arrival, the animals were randomized and housed four per cage under strictly controlled environmental conditions (22–25°C and 48–52% relative humidity) with a 12 h light/dark cycle at an intensity of 150 to 300 Lux. All mice were provided food and water *ad libitum* and were then maintained during this study.

In the intravenous study of LDD1937, 10 mg/kg of LDD1937 (dissolved in distilled water at a concentration of 2 mg/ml) were intravenously injected into the mice through the tail vein. A blood sample of approximately 0.12 ml was collected by cardiac puncture. Additionally, in the oral study of LDD1937, 10 mg/kg of LDD1937 (dissolved in the same solution used in the intravenous study) were orally administered to the mice. The analysis of LDD1937 and LDD1940, a metabolite of LDD1937, in biological samples was done with LC-MS/MS using the Waters UPLC-XEVO TQ system (Waters Corporation, Milford, CT, USA). LDD1937, LDD1940, and carbamazepine (an internal standard, IS) were ionized in the multiple reaction monitoring mode with an ESI interface for positive ions ([M+H]^+^). The turbo ion-spray interface was operated at an ion capillary voltage of 3.0 kV with a desolvation gas flow of 650 l/h and a cone gas flow of 10 l/h at 350°C. The mass transitions for LDD1937, LDD1940, and IS were *m/z* 448.17 → 113.03 (collision energy of 23 V), *m/z* 434.23 → 112.97 (collision energy of 25 V), and 237.07 → 194.04 (collision energy of 20 V), respectively. Separation was done on a reverse-phase C_18_ column (BEH, 1.7 ×100 mm i.d., 2.1 µm particle size; Waters, Ireland) with a mobile phase, distilled water containing 0.1% formic acid and acetonitrile, at 30°C. After measuring the concentrations of the LDD1937 and LDD1940, standard methods were used to calculate the pharmacokinetic parameters using a non-compartmental analysis (WinNonlin 2.1; Pharmasight Corp., Mountain View, CA, USA).

### Mouse tumor xenograft

MV-4-11 cells were inoculated subcutaneously in the flank of female BALB/c *nu/nu* (athymic nude) mice (5 × 10^6^ cells per mouse). When the tumor reached a mean volume of 100 mm^3^ (approximately 14 days after inoculation), the mice were randomly divided into three groups (*n* = 10 for the control group and *n* = 6 for the 5 mg/kg and 10 mg/kg groups) and injected with 20 ml/kg of 5 mg/kg or 10 mg/kg LDD1937 in PBS, or pure PBS (control) in the tail vein. The drug or the control PBS was administered daily for a duration of 21 days. Tumor sizes were measured twice a week for 21 days, and the tumor volumes were calculated with the following formula: *V* (volume) = *X* (length) × *D* (width)^2^/2. After 21 days, the mice were sacrificed, and the tumor weights were measured.

## SUPPLEMENTARY MATERIALS FIGURES AND TABLES



## Abbreviations

AML: acute myeloid leukemia
ATP: adenosine triphosphate
AUC: area under the plasma drug concentration-time curve
BCA: bicinchoninic acid
BSA: bovine serum albumin
CI: combination index
DMEM: Dulbecco Modified Eagle Medium
DMSO: dimethylsulfoxide
DTT: Dithiothreitol
ESI: electrospray ionization
FLT3: FMS-like receptor tyrosine kinase-3
GI50: growth inhibition of 50%
HEPES: (4-(2-hydroxyethyl)-1-piperazineethanesulfonic acid
HTRF: homogeneous time-resolved fluorescence
ICR: Imprinting Control Region
IC_50_: half maximal inhibitory concentration
IMDM: Iscove’s Modified Dulbecco’s Medium
IRAK4: interleukin-1 receptor-associated kinase 4
ITD: internal tandem duplication
Jak2: janus kinase 2
Jak3: janus kinase 3
LC-MS/MS: liquid chromatography-tandem mass spectrometry
MAPK: mitogen-activated protein kinase
OD: optical density
PARP: poly-ADP ribose polymerase
PBS: phosphate-buffered saline
PDGFR: platelet-derived growth factor receptor
PI: propidium iodide
PI3K: phosphoinositide 3-kinase
PVDF: polyvinylidene difluoride
RTK: receptor tyrosine kinase
SDS: sodium dodecyl sulfate
STAT5: Signal transducer and activator of transcription 5
TR-FRET: time-resolved fluorescence resonance energy transfer
TUNEL: terminal deoxynucleotidyl transferase dUTP nick end labeling
