# Correction: Discovery of a FLT3 inhibitor LDD1937 as an anti-leukemic agent for acute myeloid leukemia

**DOI:** 10.18632/oncotarget.25141

**Published:** 2018-04-13

**Authors:** Hyo Jeong Lee, Jungeun Lee, Pyeonghwa Jeong, Jungil Choi, Juhwa Baek, Su lin Ahn, Yeongyu Moon, Jeong Doo Heo, Young Hee Choi, Young-Won Chin, Yong-Chul Kim, Sun-Young Han

**Affiliations:** ^1^ College of Pharmacy and Research Institute of Pharmaceutical Sciences, Gyeongsang National University, Jinju, Republic of Korea; ^2^ School of Life Sciences,Gwangju Institute of Science and Technology, Gwangju, Republic of Korea; ^3^ Biomedical Science and Engineering, Gwangju Institute of Science and Technology, Gwangju, Republic of Korea; ^4^ Gyeongnam Department of Environment Toxicology and Chemistry, Korea Institutes of Toxicology, Jinju, Republic of Korea; ^5^ College of Pharmacy and BK21PLUS R-FIND Team, Dongguk University-Seoul, Goyang, Republic of Korea

**This article has been corrected:** The correct author affiliations citations are given below:

Hyo Jeong Lee^1,*^, Jungeun Lee^2,*^, Pyeonghwa Jeong^3,*^, Jungil Choi^4^, Juhwa Baek^1^, Su Jin Ahn^1^, Yeongyu Moon^4^, Jeong Doo Heo^4^, Young Hee Choi^5^, Young-Won Chin^5^, Yong-Chul Kim^2,3^ and Sun-Young Han^1^

Original article: Oncotarget. 2018; 9:924-936. https://doi.org/10.18632/oncotarget.23221

