# Supplementary material for: Discovery of a FLT3 inhibitor LDD1937 as an anti-leukemic agent for acute myeloid leukemia
Source: Oncotarget. 2017 Dec 14;9(1):924–36. doi: 10.18632/oncotarget.23221 (PMC5787524; doi:10.18632/oncotarget.23221)
Supplement: Supplementary file 1 [file oncotarget-09-924-s001.pdf]

Discovery of a FLT3 inhibitor LDD1937 as an anti-leukemic agent for acute myeloid leukemia

SUPPLEMENTARY MATERIALS

Supplementary Results

Recently, we found that 5-carboxy indirubin derivative 7 potently inhibits FLT3 kinase (IC_{50} = 8 nM) with the lack of an anti-proliferative activity against MV-4-11 which is a human leukemia cell line expressing FLT3-ITD (Supplementary Table 1). Interestingly, the corresponding 5-carboxy ester analogue 1 showed a potent anti-proliferative activity against MV-4-11 cells (IC_{50} = 41 nM), despite its very weak FLT3 inhibitory activity. Therefore, we have tried optimization to increase the inhibitory activity against both the FLT3 kinase and the growth of MV-4-11 by further derivatization at the 3′ position of indirubin skeleton.

3′-Alkyl substitutions of indirubin oxime derivatives for other kinases have been reported to provide advantages such as a higher potency and water solubility. Because there are no reports of 3′-alkyl substituted indirubin oxime derivatives with 5-carboxylic acid or ester groups as FLT3 inhibitors, we synthesized such analogs to achieve the benefits of alkyl substitutions at the 3′ oxime position. The inhibitory effects of the 3′-substituted indirubin analogs are described in Supplementary Table 1. In general, the alkyl substituted compounds showed increased inhibitory activities against both FLT3 and MV-4-11 compared to compounds 1 and 7. The analogues of the 5-ester substituted compound 1 showed potent inhibitory activities against the MV-4-11 cells as well as FLT3 kinase. However, the 5-carboxy derivatives (9–12) showed slightly lower inhibitory activities compared to the 5-ester analogues (3–6) even though they had potent inhibitory activities on FLT3 kinase. The FLT3 and MV-4-11 inhibition effects were dependent on the substituent at the R position. The FLT3 inhibitory activities increased in the order ethyl bromide and morpholine < N-methyl piperazine < amine, piperazine. The inhibitory activities of the substituents at the R position against MV-4-11 increased in the following order: ethyl bromide and morpholine < N-methyl piperazine, amine < piperazine. The ethyl bromide and morpholine substitutions at the R position (compound 2, 6, 8 and 12) did not have a crucial impact on inhibitory abilities against FLT3 and MV-4-11. The ethyl piperazine substituted compound 4 and 10 showed the most potent inhibitory activities against FLT3 (IC_{50} = 3 and 2.5 nM) and MV-4-11 (IC_{50} = 1 and 40 nM) in the each series. Moreover, compound 4 inhibited the growth of MV-4-11 cells more potently than that of CEP-701 which is a well-known FLT3 inhibitor.

Surprisingly, all the alkyl substituted series of the 5-methyl ester compound 1 showed dramatically increased inhibitory activities against on FLT3 kinase. The ethyl piperazine substituted LDD1937 showed the most potent activities against both FLT3 (IC_{50} = 3 nM) and MV-4-11 (IC_{50} = 1 nM) each with 1000- and 40-fold enhancing activities compared to the initial compound 1. Additionally, introduction of alkyl substituents at the 3′- oxime position of 5-carboxylic acid indirubin 7 greatly improved the inhibition effects against MV-4-11. Similar to the 5-methyl ester series, piperazine substituted compound 10 showed the most potent inhibitory effects against both FLT3 and MV-4-11. However, compound 10 showed only a moderate inhibitory activity against the MV-4-11 cells even though they had a potent inhibitory activity against FLT3 kinase. We expect that the 5-carboxylic acid derivatives are too polar to penetrate the cell membrane. To confirm this expectation, the permeability of the 5-carboxylic acid compounds should be checked.

Among the 13 compounds, compound 4 (LDD1937) (Figure1A), methyl (2Z,3E)-2′-oxo-3-((2-(piperazin-1-yl)ethoxy)imino)-[2,3′-biindolinylidene]-5′-carboxylate dihydrochloride, was selected and further characterized. As shown in Fig 1B, the IC_{50} of 4 (LDD1937) against the FLT3 kinase activity was 3 nM. The IC_{50}s against other kinase activities were also measured (Table 1). There is at least a 170-fold difference in the IC_{50} between FLT3 and the other kinases.

Analytical data of compounds

Methyl (2Z,3E)-3-(hydroxyimino)-2′-oxo-[2,3′-biindolinylidene]-5′-carboxylate (1)

^{1}H NMR (400 MHz, DMSO-*d6*) δ ppm 11.76–11.80 (m, 1 H), 11.05–11.10 (m, 1 H), 9.17–9.21 (m, 1 H), 8.24 (d, *J* = 8.01 Hz, 1H), 7.73–7.78 (m, 1H), 7.36–7.42 (m, 2H), 7.00–7.06 (m, 1H), 6.92–6.98 (m, 1H), 3.79–3.86 (m, 3H).

Methyl (2Z,3E)-3-((2-bromoethoxy)imino)-2′-oxo-[2,3′-biindolinylidene]-5′-carboxylate (2)

^{1}H NMR (400 MHz, DMSO-*d6*) δ ppm 11.68 (s, 1H), 11.18 (s, 1H), 9.36 (d, *J* = 1.6 Hz, 1H), 8.23 (d, *J* = 7.6 Hz, 1H), 7.83 (dd, *J* = 8.0, 1.6 Hz, 1H), 7.48 (m, 2H), 7.10 (m, 1H), 7.01 (d, *J* = 8.0 Hz, 1H), 4.98 (t, *J* = 5.6 Hz, 2H), 4.06 (t, *J* = 5.6 Hz, 2H), 3.86 (s, 3H).

Methyl (2Z,3E)-3-((2-aminoethoxy)imino)-2′-oxo-[2,3′-biindolinylidene]-5′-carboxylate hydrochloride (3)

^{1}H NMR (400 MHz, DMSO-*d6*) δ ppm 11.66 (s, 1H), 11.18 (s, 1H), 9.30 (d, *J* = 1.60 Hz, 1H), 8.24 (d, *J* = 7.78 Hz, 1H), 8.17 (br s, 2H), 7.80 (dd, *J* = 8.13, 1.72 Hz, 1H), 7.41–7.48 (m, 2H), 7.04 (ddd, *J* = 7.90, 5.72, 2.63 Hz, 1H), 6.98 (d, *J* = 8.01 Hz, 1H), 4.78–4.85 (m, 2H), 3.79–3.86 (m, 3H), 3.47 (d, *J* = 4.81 Hz, 2H).

Methyl (2Z,3E)-2′-oxo-3-((2-(piperazin-1-yl)ethoxy) imino)-[2,3′-biindolinylidene]-5′-carboxylate dihydrochloride (4, LDD1937)

^{1}H NMR (400 MHz, DMSO-*d6*) δ ppm 11.70 (s, 1H), 11.23 (s, 1H), 9.36 (s, 1H), 8.27 (d, *J* = 7.6 Hz, 1H), 7.84 (d, *J* = 8.4 Hz, 1H), 7.48 (m, 2H), 7.07 (m, 1H), 7.02 (d, *J* = 8.8 Hz, 1H), 5.03 (m, 2H), 3.88 (s, 3H), 3.30 (m, 10H, overlapped with DMSO).

Methyl (2Z,3E)-3-((2-(4-methylpiperazin-1-yl)ethoxy) imino)-2′-oxo-[2,3′-biindolinylidene]-5′-carboxylate dihydrochloride (5)

^{1}H NMR (400 MHz, DMSO-*d6*) δ ppm 11.71 (s, 1H), 11.22 (s, 1H), 9.38 (d, *J* = 1.6 Hz, 1H), 8.24 (d, *J* = 7.6 Hz, 1H), 7.84 (dd, *J* = 8.4, 1.6 Hz, 1H), 7.48 (m, 2H), 7.08 (m, 1H), 7.02 (d, *J* = 8.4 Hz, 1H), 4.96 (m, 2H), 3.87 (s, 3H), 3.30 (m, 10H, overlapped with water).

Methyl (2Z,3E)-3-((2-morpholinoethoxy)imino)-2′-oxo-[2,3′-biindolinylidene]-5′-carboxylate hydrochloride (6)

^{1}H NMR (400 MHz, DMSO-*d6*) δ 11.70 (s, 1H), 11.22 (s, 1H), 10.94 (br s, 1H, morpholine N^{+}-H), 9.36 (s, 1H), 8.26 (d, *J* = 7.6 Hz, 1H), 7.84 (dd, *J* = 8.0, 2.0 Hz, 1H), 7.49 (m, 2H), 7.09 (m, 1H), 7.02 (d, *J* = 8.0 Hz, 1H), 5.08 (br s, 2H), 3.97 (m, 2H), 3.80 (s, 3H), 3.81 (m, 4H), 3.56 (m, 2H), 3.18 (m, 2H, partially overlapped with water).

(2Z,3E)-3-(hydroxyimino)-2′-oxo-[2,3′-biindolinylidene]-5′-carboxylic acid (7)

^{1}H NMR (400 MHz, DMSO-*d6*) δ ppm 11.68 (s, 1H), 11.23 (s, 1H), 8.73–8.78 (m, 1H), 8.18 (s, 1H), 8.01 (d, *J* = 5.04 Hz, 1H), 7.49 (m, 2H), 6.95 (d, *J* = 8.0 Hz, 1H).

(2Z,3E)-3-((2-bromoethoxy)imino)-2′-oxo-[2,3′-biindolinylidene]-5′-carboxylic acid (8)

^{1}H NMR (400 MHz, DMSO-*d6*) δ ppm 11.66 (s, 1H), 11.11 (s, 1H), 9.34 (s, 1H), 8.22 (d, *J* = 7.6 Hz, 1H), 7.80 (d, *J* = 8.0 Hz, 1H), 7.63 (m, 2H), 7.08 (m, 1H), 6.97 (d, *J* = 8.4 Hz, 1H), 4.96 (t, *J* = 5.6 Hz, 2H), 4.03 (t, *J* = 5.6 Hz, 2H).

(2Z,3E)-3-((2-aminoethoxy)imino)-2′-oxo-[2,3′-biindolinylidene]-5′-carboxylic acid hydrochloride (9)

^{1}H NMR (400 MHz, DMSO-*d6*) δ ppm 11.68 (s, 1H), 11.18 (s, 1H), 9.35 (d, *J* = 1.2 Hz, 1H), 8.26 (d, *J* = 7.6 Hz, 1H), 8.17 (s, 3H, H^{+}), 7.82 (dd, *J* = 8.0, 1.6 Hz, 1H), 7.48 (m, 2H), 7.08 (m, 1H), 7.00 (d, *J* = 8.4 Hz, 1H), 4.83 (t, *J* = 5.2 Hz, 2H), 3.45 (t, *J* = 5.2 Hz, 2H).

(2Z,3E)-2′-oxo-3-((2-(piperazin-1-yl)ethoxy)imino)-[2,3′-biindolinylidene]-5′-carboxylic acid dihydrochloride (10, LDD1940)

^{1}H NMR (400 MHz, DMSO-*d6*) δ ppm 11.69 (s, 1H), 11.17 (s, 1H), 9.37 (s, 1H), 8.91 (br s, 2H, piperazine N^{+}-H), 8.23 (d, *J* = 7.6 Hz, 1H), 7.82 (d, *J* = 8.4 Hz, 1H), 7.47 (m, 2H), 7.07 (m, 1H), 7.00 (d, *J* = 8.4 Hz, 1H), 4.92 (br s, 2H), 3.05 (m, 10H, overlapped with water).

(2Z,3E)-3-((2-(4-methylpiperazin-1-yl)ethoxy)imino)-2′-oxo-[2,3′-biindolinylidene]-5′-carboxylic acid dihydrochloride (11)

^{1}H NMR (400 MHz, DMSO-*d6*) δ ppm 11.68 (s, 1H), 11.16 (s, 1H), 9.36 (s, 1H), 8.23 (d, *J* = 8.0 Hz, 1H), 7.82 (d, *J* = 8.0 Hz, 1H), 7.47 (m, 2H), 7.07 (m, 1H), 6.99 (d, *J* = 8.0 Hz, 1H), 4.93 (br s, 2H), 3.17 (m, 10H, overlapped with water), 2.77 (s, 3H).

(2Z,3E)-3-((2-morpholinoethoxy)imino)-2′-oxo-[2,3′-biindolinylidene]-5′-carboxylic acid hydrochloride (12)

^{1}H NMR (400 MHz, DMSO-*d6*) δ ppm 11.67(s, 1H), 11.27 (br s, 1H, morpholine N^{+}-H), 11.18 (s, 1H), 9.34 (s, 1H), 8.25 (d, *J* = 7.6 Hz, 1H), 7.82 (d, *J* = 8.4 Hz, 1H), 7.48 (m, 2H), 7.08 (m, 1H), 6.99 (d, *J* = 8.0 Hz, 1H), 5.06 (br s, 2H), 3.96 (m, 2H), 3.80 (m, 4H), 3.53 (m, 2H), 3.24 (m, 2H, partially overlapped with water).

Ethyl (E)-4-(2-(hydroxyimino)acetamido)benzoate (13)

^{1}H NMR (400 MHz, DMSO-*d6*) δ ppm 12.28 (br s, 1H), 10.49 (s, 1H), 7.87–7.92 (m, 2H), 7.77–7.82 (m, 2H), 4.25 (q, *J* = 7.17 Hz, 2H), 1.27 (t, *J* = 7.10 Hz, 3H).

2,3-dioxoindoline-5-carboxylic acid (14)

^{1}H NMR (400 MHz, DMSO-*d6*) δ ppm 11.35 (s, 1H), 8.10 (dd, *J* = 8.24, 1.60 Hz, 1H), 7.87 (d, *J* = 1.60 Hz, 1H), 6.96 (d, *J* = 8.24 Hz, 1H).

Methyl 2,3-dioxoindoline-5-carboxylate (15)

^{1}H NMR (400 MHz, DMSO-*d6*) δ ppm 8.12 (d, *J* = 8.24 Hz, 1H), 7.87–7.91 (m, 1H), 6.97 (dd, *J* = 8.36, 1.49 Hz, 1H), 3.77–3.82 (m, 3H).

methyl (Z)-2′,3-dioxo-[2,3′-biindolinylidene]-5′-carboxylate (16)

^{1}H NMR (400 MHz, DMSO-*d6*) δ ppm 11.25–11.30 (m, 1H), 11.09–11.14 (m, 1H), 9.42–9.47 (m, 1H), 7.87–7.93 (m, 1H), 7.66–7.73 (m, 1H), 7.60 (ddd, *J* = 8.19, 7.16, 1.37 Hz, 1H), 7.41–7.47 (m, 1H), 7.03–7.08 (m, 1H), 6.98–7.02 (m, 1H), 3.78–3.93 (m, 3H).

methyl (2Z,3E)-3-((2-((tert-butoxycarbonyl)amino) ethoxy)imino)-2′-oxo-[2,3′-biindolinylidene]-5′-carboxylate (17):

^{1}H NMR (400 MHz, DMSO-*d6*) δ ppm 11.63 (br s, 1H), 9.37 (d, *J* = 1.37 Hz, 1H), 8.19 (d, *J* = 7.78 Hz, 1 H), 7.78 (dd, *J* = 8.13, 1.72 Hz, 1H), 7.41 (d, *J* = 3.89 Hz, 2H), 7.09 (s, 1H), 6.99 (br s, 1H), 6.96 (d, *J* = 8.01 Hz, 1H), 4.63 (t, *J* = 4.81 Hz, 2H), 3.81 (s, 3H), 3.52 (d, *J* = 5.27 Hz, 2H), 1.29 (s, 9H).

(2Z,3E)-3-((2-((tert-butoxycarbonyl)amino)ethoxy) imino)-2′-oxo-[2,3′-biindolinylidene]-5′-carboxylic acid (18)

^{1}H NMR (400 MHz, DMSO-*d6*) δ ppm 11.62 (s, 1H), 11.07 (s, 1H), 9.35 (s, 1H), 8.17 (d, *J* = 7.56 Hz, 1 H), 7.76 (dd, *J* = 8.13, 1.26 Hz, 1H), 7.41 (d, *J* = 3.66 Hz, 2H), 7.02–7.08 (m, 1H), 6.99 (dt, *J* = 7.73, 4.04 Hz, 1H), 6.93 (d, *J* = 8.01 Hz, 1H), 4.60 (t, *J* = 4.58 Hz, 2H), 3.48–3.53 (m, 2H), 1.22–1.30 (m, 9H).

Methyl (2Z,3E)-2′-oxo-3-((2-(piperazin-1-yl)ethoxy) imino)-[2,3′-biindolinylidene]-5′-carboxylate (19)

^{1}H NMR (400 MHz, DMSO-*d6*) δ ppm 11.70 (br s, 1H), 11.18 (br s, 1H), 9.41 (d, *J* = 1.6 Hz, 1H), 8.18 (d, *J* = 7.6 Hz, 1H), 7.82 (dd, *J* = 8.0, 1.6 Hz, 1H), 7.46 (m, 2H), 7.05 (m, 1H), 7.00 (d, *J* = 8.0 Hz, 1H), 4.81 (t, *J* = 5.6 Hz, 2H), 3.85 (s, 3H), 2.87 (t, *J* = 5.6 Hz, 2H), 2.67 (m, 4H), 2.44 (m, 4H, partially overlapped with DMSO).

Methyl (2Z,3E)-3-((2-(4-methylpiperazin-1-yl)ethoxy) imino)-2′-oxo-[2,3′-biindolinylidene]-5′-carboxylate (20)

^{1}H NMR (400 MHz, DMSO-*d6*) δ ppm 11.70 (br s, 1H), 11.17 (br s, 1H), 9.41 (d, *J* = 1.6 Hz, 1H), 8.18 (d, *J* = 7.6 Hz, 1H), 7.82 (dd, *J* = 8.0, 1.6 Hz, 1H), 7.46 (m, 2H), 7.08 (m, 1H), 7.00 (d, *J* = 8.0 Hz, 1H), 4.81 (t, *J* = 5.6 Hz, 2H), 3.85 (s, 3H), 2.91 (t, *J* = 5.6 Hz, 2H), 2.55 (br s, 4H, overlapped with water), 2.31 (br s, 4H), 2.10 (s, 3H).

Methyl (2Z,3E)-3-((2-morpholinoethoxy)imino)-2′-oxo-[2,3′-biindolinylidene]-5′-carboxylate (21)

^{1}H NMR (400 MHz, DMSO-*d6*) δ ppm 11.70(br s, 1H), 11.17 (br s, 1H), 9.41 (d, *J* = 1.6 Hz, 1H), 8.20 (d, *J* = 8.0 Hz, 1H), 7.83 (dd, *J* = 8.4, 1.6 Hz, 1H), 7.46 (m, 2H), 7.09 (m, 1H), 7.00 (d, *J* = 8.4 Hz, 1H), 4.83 (t, *J* = 5.6 Hz, 2H), 3.85 (s, 3H), 3.57 (t, *J* = 4.8 Hz, 2H), 2.92 (t, *J* = 5.6 Hz, 2H), 2.52 (t, 4H, partially overlapped with DMSO).

Supplementary Table 1: Structure activity relationship of indirubin derivatives

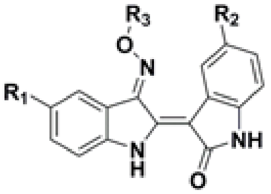

Compound	R_1	R_2	R_3	IC_{50} (nM)	GI_{50} (nM)
				**FLT3[a]**	**MV-4-11**
**1**	H	$COOCH_3$	H	1215 ± 36.12	41 ± 7
**2**	H	$COOCH_3$	CH_2CH_3Br	19.2 ± 1.88	51 ± 11
**3**	H	$COOCH_3$	$CH_2CH_2NH_2 \cdot HCL$	4.5 ± 0.75	15 ± 3
LDD1937 (**4**)	H	$COOCH_3$	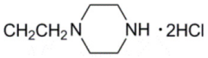	3 ± 0.53	1 ± 0.15
**5**	H	$COOCH_3$	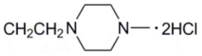	10.78 ± 2.02	11 ± 1.5
**6**	H	$COOCH_3$	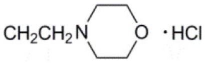	252.9 ± 81.41	53 ± 11
**7**	H	COOH	H	24.98 ± 3.32	>1,000
**8**	H	COOH	CH_2CH_2Br	12.93 ± 0.97	340 ± 81
**9**	H	COOH	$CH_2CH_2NH_2 \cdot HCL$	3.3 ± 0.11	150 ± 33
LDD1940 (**10**)	H	COOH	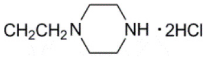	2.45 ± 0.42	40 ± 12
**11**	H	COOH	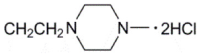	3.3 ± 0.6	176 ± 51
**12**	H	COOH	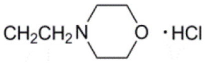	7.55 ± 1.01	>1,000

The data indicate the mean ± S.D

Supplementary Table 2: Anti-proliferative activities of LDD1940 against various cancer cell lines.

**Cell line**	**IC_{50} (μM)**
MV-4-11	0.04 ± 0.012
Jurkat	>10
PC-3	>10
MCF-7	>10
K562	>10

Growth inhibition of tumor cell lines was measured using the EZ-Cytox Cell Viability Assay kit. GI_{50}s were calculated by a non-linear regression.
The data indicate the mean ± S.D

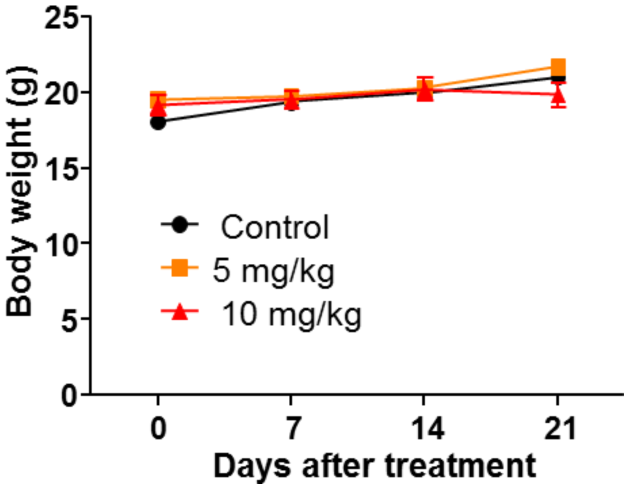

Supplementary Figure 1: Body weight changes of the mice after LDD1937 treatment. All results represent the mean ± SEM.

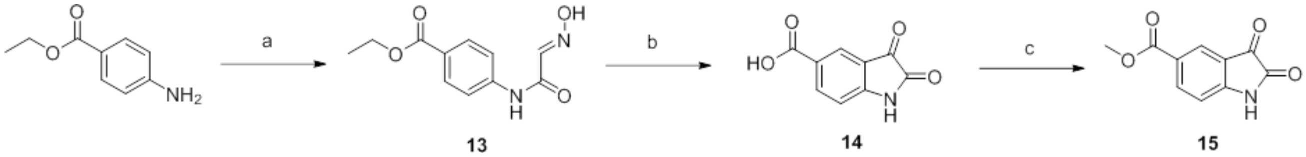

Supplementary Figure 2: Synthetic scheme of compounds 13, 14 and 15. Compound 13 was synthesized by the reaction of chloral hydrate with ethyl 4-aminobenzoate in water/H_2SO_4 mixture. And then compound 14 was obtained through simple cyclization in the presence of concentrated H_2SO_4. Compound 15 was prepared by Fischer esterification with H_2SO_4 in methanol. Reagents and conditions: (a) chloral hydrate, Na_2SO_4, water, H_2SO_4, 100°C, on; (b) Conc. H_2SO_4, 90°C, 25min; (c) H_2SO_4, 60°C, 1 hr.

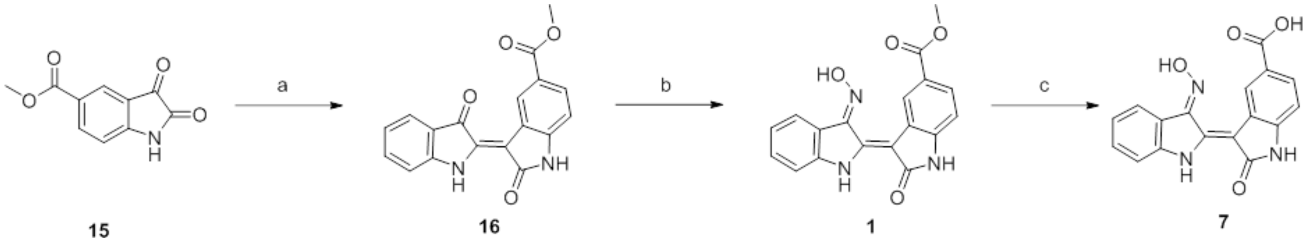

Supplementary Figure 3: Synthetic scheme of compounds 1, 7 and 16. Compound 16 was prepared by condensation of appropriately isatin, iv, with indoxyl acetate in the presence of Na_2CO_3 in methanol. Indirubin-3′-oxime 1 was prepared from reaction of indirubin with hydroxylamine in pyridine. Compound 7 was obtained by simple hydrolysis reaction with 1N NaOH in methanol and acetone. Reagents and conditions: (a) indoxyl acetate, Na_2CO_3, MeOH, RT, on; (b) $H_2NOH{\cdot}HCl$, pyridine, reflux, on; (c) NaOH, MeOH, acetone, RT, 30 min.

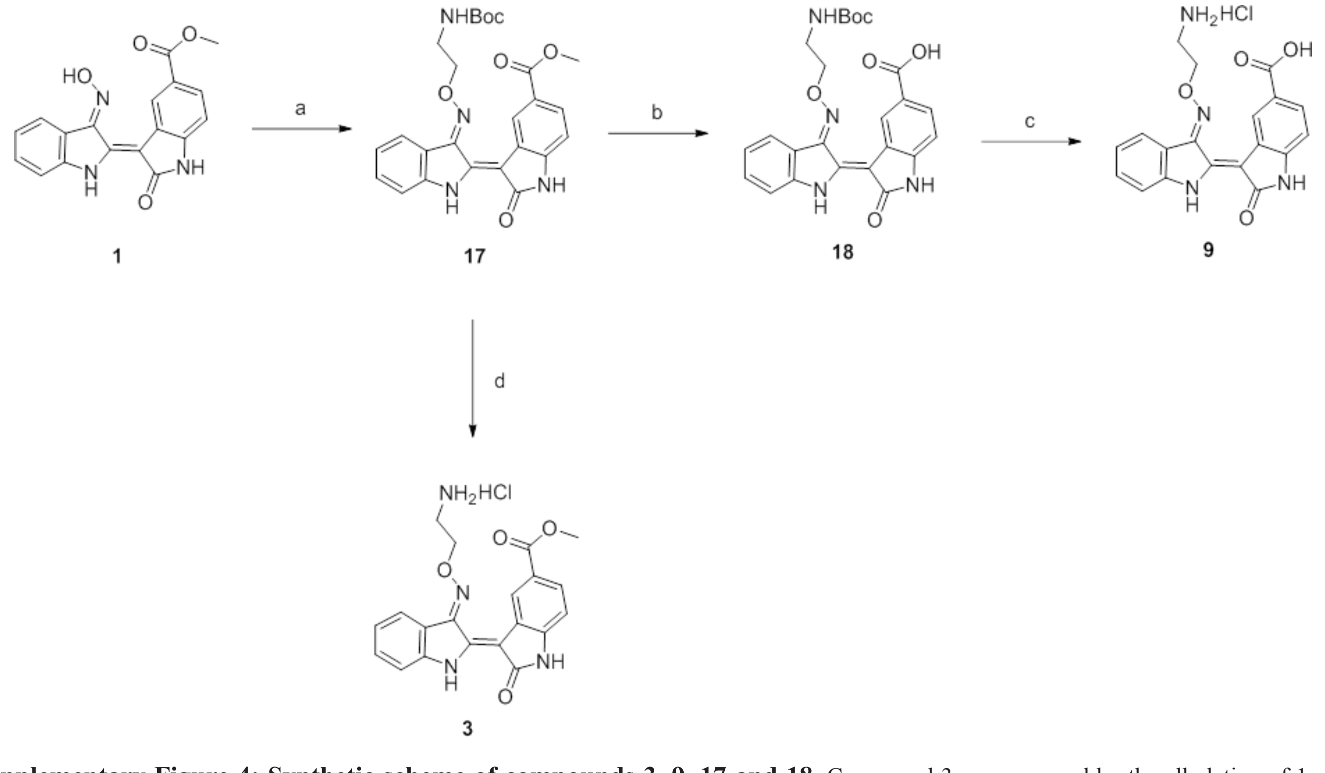

Supplementary Figure 4: Synthetic scheme of compounds 3, 9, 17 and 18. Compound 3 was prepared by the alkylation of 1 with 2-(Boc-amino)ethyl bromide, 17, followed by Boc deprotection reaction using 4N hydrochloric acid solution. Compound 9 was prepared by hydrolysis reaction using 1N NaOH solution of the corresponding ester compound, 18, and treated with 4N hydrochloric acid solution. Reagents and conditions: (a) 2-(Boc-amino)ethyl bromide, K_2CO_3, DMF, rt, on; (b) 1N NaOH, 1,4-dioxane, 40°C, on; (c) 4N HCl in 1,4-dioxane, THF, 0°C, 1 h; (d) 4N HCl in 1,4-dioxane, MC, 0°C, 30 min.

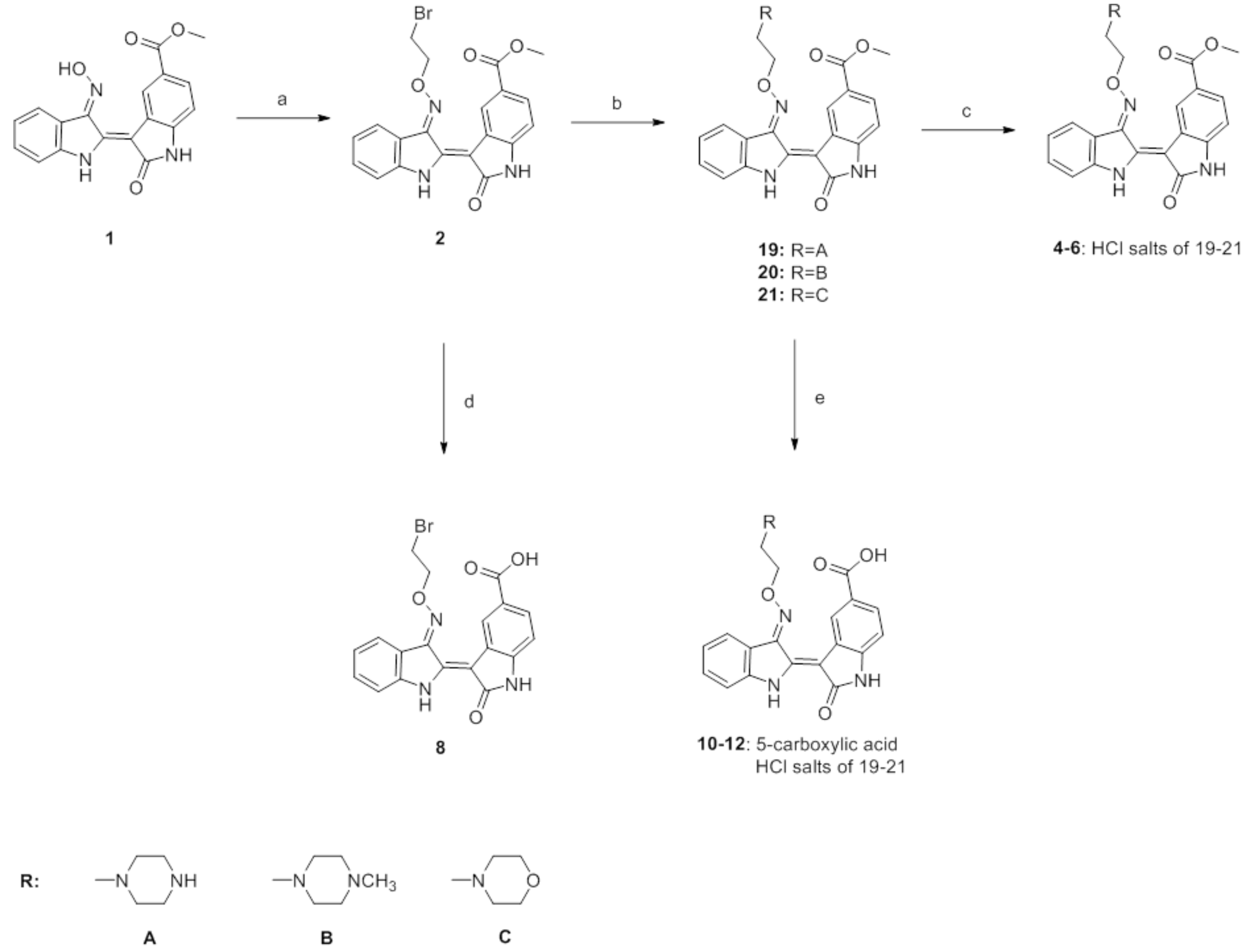

Supplementary Figure 5: Synthetic scheme of compounds 2, 4-6, 8 and 10-12. 3′-Substituted oximes were obtained through the simple two step reactions. The alkyl bromide intermediate (2) was synthesized by the reaction of 1 with 1,2-dibromoethane in DMF and Et_3N at room temperature. And then compounds (19-21) were obtained by the reaction of ethyl bromide intermediate with the appropriate amines: piperazine, N-methyl piperazine and morpholine. The corresponding salts of 4-6 were formed with 4N HCl in 1,4-dioxane. 5-Carboxylic acid derivatives 8 was prepared by hydrolysis reaction of the corresponding ester compounds. Compounds (10-12) were prepared by consecutive reaction. The compounds (19-21) were hydrolyzed by 1N NaOH solution and neutralized by 1N HCl solution. After that, filtered using methanol and obtained products were treated with 4N hydrochloric acid solution. Reagents and conditions: (a) 1,2-dibromoethane, Et_3N, DMF, RT, on; (b) amines A-C, DMF, 50°C, on; (c) 4N HCl in 1,4-dioxane, THF, 0°C, 30 min; (d) 1N NaOH, 1,4-dioxane, 40°C, on; (e) 1N NaOH, 1,4-dioxane, MeOH, 40°C, on, 4N HCl in dioxane, MC, 0°C, 30 min.
